# Local perspectives on Ebola during its tenth outbreak in DR Congo: A nationwide qualitative study

**DOI:** 10.1371/journal.pone.0241120

**Published:** 2020-10-22

**Authors:** Basilua Andre Muzembo, Ngangu Patrick Ntontolo, Nlandu Roger Ngatu, Januka Khatiwada, Kabamba Leon Ngombe, Oscar Luboya Numbi, Kabamba Michel Nzaji, Kabinda Jeff Maotela, Mukonkole Jean Ngoyi, Tomoko Suzuki, Koji Wada, Shunya Ikeda

**Affiliations:** 1 Department of Public Health, School of Medicine, International University of Health and Welfare, Narita, Japan; 2 Graduate School of Medicine, Dentistry and Pharmaceutical Sciences, Okayama University, Okayama, Japan; 3 Department of Family Medicine and Primary health, Protestant University of Congo, Kinshasa, DR Congo; 4 Institut Médical Evangélique (IME), Kimpese, DR Congo; 5 Department of Public Health, Kagawa University Faculty of Medicine, Miki, Kagawa, Japan; 6 Department of Public Health, University of Kamina, Kamina, DR Congo; 7 School of Public Health, University of Lubumbashi, Lubumbashi, DR Congo; 8 Research Unit of National Vaccination Program, Ministry of Health, Kinshasa, DR Congo; 9 Centre National de Transfusion Sanguine, Kinshasa, DR Congo; 10 Research Unit, ISTM-Lubumbashi, Lubumbashi, DR Congo; University of New South Wales, AUSTRALIA

## Abstract

**Background:**

The Democratic Republic of Congo (DR Congo) struggled to end the tenth outbreak of Ebola virus disease (Ebola), which appeared in North Kivu in 2018. It was reported that rumors were hampering the response effort. We sought to identify any rumors that could have influenced outbreak containment and affected prevention in unaffected areas of DR Congo.

**Methods:**

We conducted a qualitative study in DR Congo over a period of 2 months (from August 1 to September 30, 2019) using in-depth interviews (IDIs) and focus group discussions (FGDs). The participants were recruited from five regional blocks using purposeful sampling. Both areas currently undergoing outbreaks and presently unaffected areas were included. We collected participants’ opinions, views, and beliefs about the Ebola virus. The IDIs (n = 60) were performed with key influencers (schoolteachers, religious and political leaders/analysts, and Ebola-frontline workers), following a semi-structured interview guide. FGDs (n = 10) were conducted with community members. Interviews were recorded with a digital voice recorder and simultaneous note-taking. Participant responses were categorized in terms of their themes and subthemes.

**Results:**

We identified 3 high-level themes and 15 subthemes (given here in parentheses): (1) inadequate knowledge of the origin or cause of Ebola (belief in a metaphysical origin, insufficient awareness of Ebola transmission via an infected corpse, interpretation of disease as God’s punishment, belief in nosocomial Ebola, poor hygiene, and bathing in the Congo River). Ebola was interpreted as (2) a plot by multinational corporations (fears of genocide, Ebola understood as a biological weapon, concerns over organ trafficking, and Ebola was taken to be the result of business actions). Finally Ebola was rumored to be subject to (3) politicization (political authorities seen as ambivalent, exclusion of some community leaders from response efforts, distrust of political authorities, and distrust in the healthcare system).

**Conclusions:**

Due to the skepticism against Ebola countermeasures, it is critical to understand widespread beliefs about the disease to implement actions that will be effective, including integrating response with the unmet needs of the population.

## Introduction

Ebola virus disease (Ebola) remains a significant public health problem in the Democratic Republic of Congo (DR Congo). From 1976 to 2019, the country recorded 11 outbreaks of the disease. The tenth of these (declared on August 1, 2018, in the Kivus) was found to be difficult to control, in spite of the efforts of national and international efforts. Some concerns have been raised about the effectiveness of the control efforts, along with the spread of rumors and distrust in the governmental authorities.

As of June 21, 2020, the Kivu epidemic had resulted in 3,470 cases of Ebola in DR Congo, with a total death toll of 2,280 [1]. The WHO declared that this outbreak was a public health emergency of international concern. This is the most deadly outbreak of Ebola in the country since its discovery in 1976 and the second deadliest in the recorded history of Ebola, following only the 2013–2016 West Africa Ebola epidemic, which recorded 11,323 deaths [2]. One element of particular concern in this outbreak is that it marks the first time that it has occurred in an area affected by civil unrest, which hampered control efforts. In this neglected and conflict-riven area, locals often distrust strangers, including foreign doctors and official authorities. This outbreak differs from previous ones in terms of the interventional tools available and the lack of security in affected areas, together with many internally displaced people living in difficult conditions.

Furthermore, Ebola treatment centers (ETCs), healthcare centers, community health workers, and healthcare workers (HCWs) have been attacked, threatened, and killed by armed assailants [3]. For example, one WHO physician was murdered during an attack on a hospital [4]. Furthermore, due to the weak healthcare system and poor roads in the region, response teams were prevented from reaching the most remote areas, where Ebola continued to rage on and spread.

Fortunately, tools were available to fight the disease; these included experimental vaccines and drugs and mobile laboratories, which had not been available in past outbreaks. However, the outbreak remained challenging to control and contain. Community participation and the confidence of the population are key factors that could lead to adequate control of the Ebola epidemic. Attempts to control the outbreak, however, were hampered by many factors, including community resistance, fear and rumors fed by political unrest and insecurity, and leadership deficits [5, 6]. In addition, a lack of trust in institutions among the population delayed the control of the outbreak [7]. Hitherto, only quantitative methods have been used to assess these contributing factors. However, to explain how and why these rumors and lack of trust exist, a qualitative assessment, including the development of an understanding of local beliefs and views, is necessary [8], as this approach can help establish the causes of population resistance with a degree of nuance. Some in Ebola-affected areas believe that the presence of humanitarian aid organizations and government representatives is a main cause for the spread of Ebola [9], that Ebola is rooted in someone’s commercial benefit [3], or that it has a supernatural origin [6]. Even though cases of Ebola in this epidemic were confined to Eastern DR Congo, the disease had the potential to spread to other provinces through the movement of people from the affected zones. It was thus thought necessary to halt movement to repress the dissemination of Ebola across the country or to the bordering countries.

Therefore, local beliefs and views that could have influenced preventive behaviors related to Ebola in unaffected areas were investigated because the risk of spread to these areas was rising. Listening to and understanding people in unaffected areas are crucial if the country to be prepared for future outbreaks and develop a response plan. In this study, we interviewed key informants, including stakeholders and gatekeepers in the study area to establish prevalent and recent rumors that may have led to community resistance in Ebola-affected areas of DR Congo and that may be affecting Ebola prevention in unaffected areas, such as in the form of vaccine refusal.

## Methods

### Ethics statement

We obtained ethical approval for this study from the Research Ethics Committee of Lubumbashi University (DR Congo; reference number UNILU/CEM/078/2019) and the Ethics Committee of the International University of Health and Welfare (IUHW, Japan; reference number 19-Im-001). All participants provided written informed consent. This study included telephone interviews. For remote participants who were interviewed by telephone, research assistants helped obtain their informed consent. Data for the present study were collected as a part of a nationwide study on knowledge, attitudes, and practices related to Ebola in DR Congo. All data are stored at the IUHW.

### Study design and setting

We conducted a descriptive qualitative study over a period of 2 months (from August 1 to September 30, 2019) in DR Congo, using in-depth-interviews (IDIs) and focus group discussions (FGDs). Narrative approach was used. This design was adopted to enable a deeper understanding of local views, feelings, and experiences regarding the Ebola outbreak. This is intended to map and debunk rumors and improve Ebola response and preparedness [8]. We used Consolidated Criteria for Reporting Qualitative Health Research for this study [10].

### Participants and procedure

The participants were recruited from five regional blocks, using a purposeful sampling technique. The entire country was divided into five blocks (East, West, North, South, and Central), following the pre-existing divisions of Bas-Congo (West), Orientale Province (North), Kasaïs (Central), Katanga (South), and the Kivus (East). Kinshasa, the capital city, was separately included due to its highly urbanized character and large population (of about 12 million), its role as a main hub for trade [11], and its high airline connectivity with Ebola zones through Goma in the Kivus [12]. We recruited participants in each regional block for the IDIs. The participants were schoolteachers, pastors and priests, political leaders/analysts, and members of response teams who were able to provide in-depth information. In addition, local community members were recruited only in Kinshasa and Bas-Congo for FGDs for feasibility reasons. Inclusion criteria for this study were aged 18 years or over, be able to provide in-depth information, local resident, and having an interest in the outbreak. Religious leaders in particular were sought after due to the high level of trust they enjoyed in their communities. Community recruitment was facilitated by community health workers after the principal investigator (BAM) presented to them the purpose of the study. Research assistants working in our quantitative study, which was carried out simultaneously with this study, also directly explained the study purpose to the participants. Chain referral was used to recruit participants. Interviews and additional recruitment were performed by BAM, KLN, and MJN. Participation in the study was voluntary, and participants were not paid for their participation. They were asked to freely share or discuss their views, beliefs, experiences, and perceptions regarding the Ebola outbreak, as well as whether they trusted the Congolese authorities. Interviews with the participants were performed face-to-face or via telephone with only the interviewer and the participant present. We asked the participants for permission to record the conversations using a digital voice recorder, and then we transcribed the conversations. This was supplemented by note-taking during the IDIs and FGDs. No information was recorded that would identify the participant. Data from Katanga were collected solely through field notes. For practical purposes, data were collected until the planned number of participants was recruited (for IDIs, n = 10 per regional block, and 10 FGDs in total). This sample size was determined from pragmatic considerations and in accordance with guidelines found in the literature [13, 14]. It was not considered obligatory to continue data collection until saturation of data was achieved [15].

### Data collection tools

We used semi-structured IDI and FGD guides as tools. To develop these guides, we followed the constructs of the Health Belief Model (HBM) [16] and a review of similar studies conducted in DR Congo [6, 7]. The HBM framework is important for predicting health-related behaviors and improving health education programs. We designed guides that contained questions in French (available in the S1 Appendix and S2 Appendix). However, participants and informants were allowed to use Lingala, a lingua franca that is commonly spoken in DR Congo. IDIs and FGDs began with the following warm-up questions. What is commonly said in DR Congo concerning health? Have you heard of the ongoing Ebola outbreak in in the eastern part? (The second question was asked in cases where the response to the first question was unsatisfactory). The remaining topic prompts asked for participants’ perception of the cause and origin of Ebola, their level of trust in the government and in HCW Ebola response teams, perceived barriers to the Ebola response, and possible solutions to stop the ongoing outbreak. The questionnaire was pretested in 20 persons from Bas-Congo (n = 10) and Kinshasa (n = 10) to test the duration of administration, comprehensibility, clarity, and ordering of the questionnaire.

### Data analysis

There were two research questions. What rumors are there that might hinder Ebola containment in the unaffected zones and in the affected zones? Do people trust the Congolese authorities?

All audio transcriptions and field notes were included in the analyses. They were reviewed and summarized by two authors who listened to the voice recordings and read the field notes. The concepts present in the recordings and notes were identified and coded, and recurring concepts were narrowed down to identify subthemes and themes. The links between themes were also assessed. Individual opinions (for example, a claim linking Ebola to a Balkanization project) that could not be placed into a category or subtheme were recorded for possible practical use by policymakers to predict future rumors. During transcription, the interviews conducted in French were translated into English by two researchers. We report quotations, subthemes, and themes in this study. Subthemes were grouped under thematic umbrellas, and quotations that linked the themes were taken into account. No political leader or analyst consented to being audio recorded. Off-the-record comments only were used to analyze their views and beliefs.

## Results

In total, 110 participants [aged 20–80 years; males (80/110; 72.7%) and females (30/110; 27.3%)] were interviewed, with 35 each in Kinshasa and Bas-Congo and 10 each in Katanga, Orientale province, Kasaïs, and the Kivus (Table 1). None of the 10 participants from the Kivus were Ebola survivors or had been directly impacted by an Ebola case.

**Table 1:** Participants included in this study.

Participants	Regional block	Total number (N = 110)
School and university teachers	Bas-Congo (7)	37
	Katanga (n = 10)	
	The Kivus (n = 5)	
	Kasaïs (n = 10)	
	Orientale province (n = 5)	
Political leaders/analysts	Bas-Congo (n = 1)	11
	Kinshasa (n = 8)	
	Orientale province (n = 2)	
Priests and pastors	Bas-Congo (n = 2)	9
	Kinshasa (n = 2)	
	The Kivus (n = 2)	
	Orientale province (n = 3)	
Ebola response team members	The Kivus (n = 3)	3
Community members	Bas-Congo (n = 25)	50
	Kinshasa (n = 25)	

**Fig 1:**
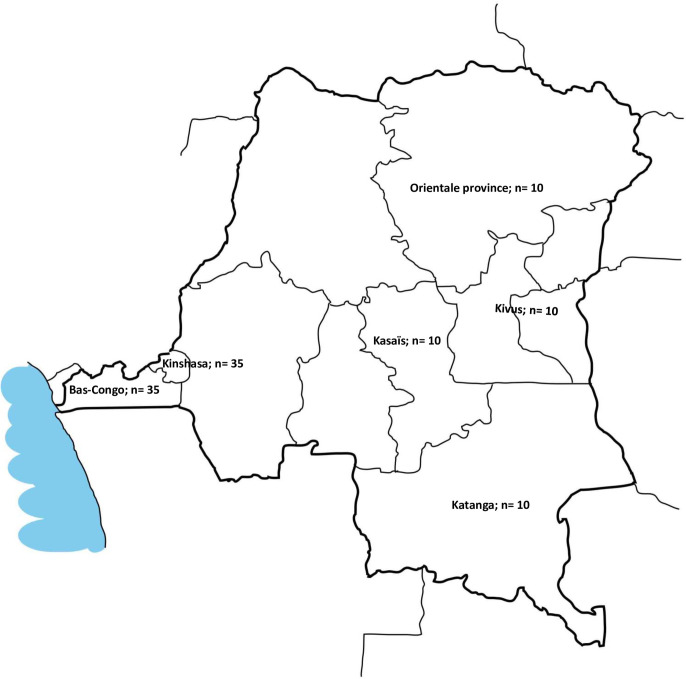
Map of DR Congo displaying the origins of the 110 participants. The participants were recruited from five regional blocks and the capital city (Kinshasa).

In all, 60 participants participated in the IDIs, and 50 in ten FGDs (with 5 participants each), with their origins marked on the map in Fig 1. Each IDI took approximately 15 to 30 minutes to complete, and the FGDs lasted 60–70 minutes.

The political leaders and analysts had predominantly negative opinions of the efforts to control Ebola at the beginning of the outbreak. They criticized the way that the response teams and the government were handling it as inadequate. One health official gave the opinion that the current outbreak may have begun in May 2018, but because the HCWs allocated to the surveillance system in the North Kivu health division were on strike due to nonpayment of salary, the information was not reported to the national health authorities. Participants in the affected areas called for Ebola responders who could speak local languages. Ebola responders from outside the Kivus were seen as outsiders by locals in the Ebola-affected areas, and they were thought to be deliberately contributing to the spread of Ebola for their own financial gain. The most frequently expressed desire from those in the unaffected areas were for the HCWs to be prepared and to implement prevention measures. Most participants considered that Ebola was only one of many problems faced by locals in the affected areas. They felt that little attention was paid to other health problems, socioeconomic challenges, and the daily needs of the affected population; their daily social priorities were left unaddressed during the response to the Ebola outbreak. Some participants reported that the government was insensitive to social and political unrest in East DR Congo.

Social media, religious leaders, and word-of-mouth were the most cited sources of rumors.

**Fig 2:**
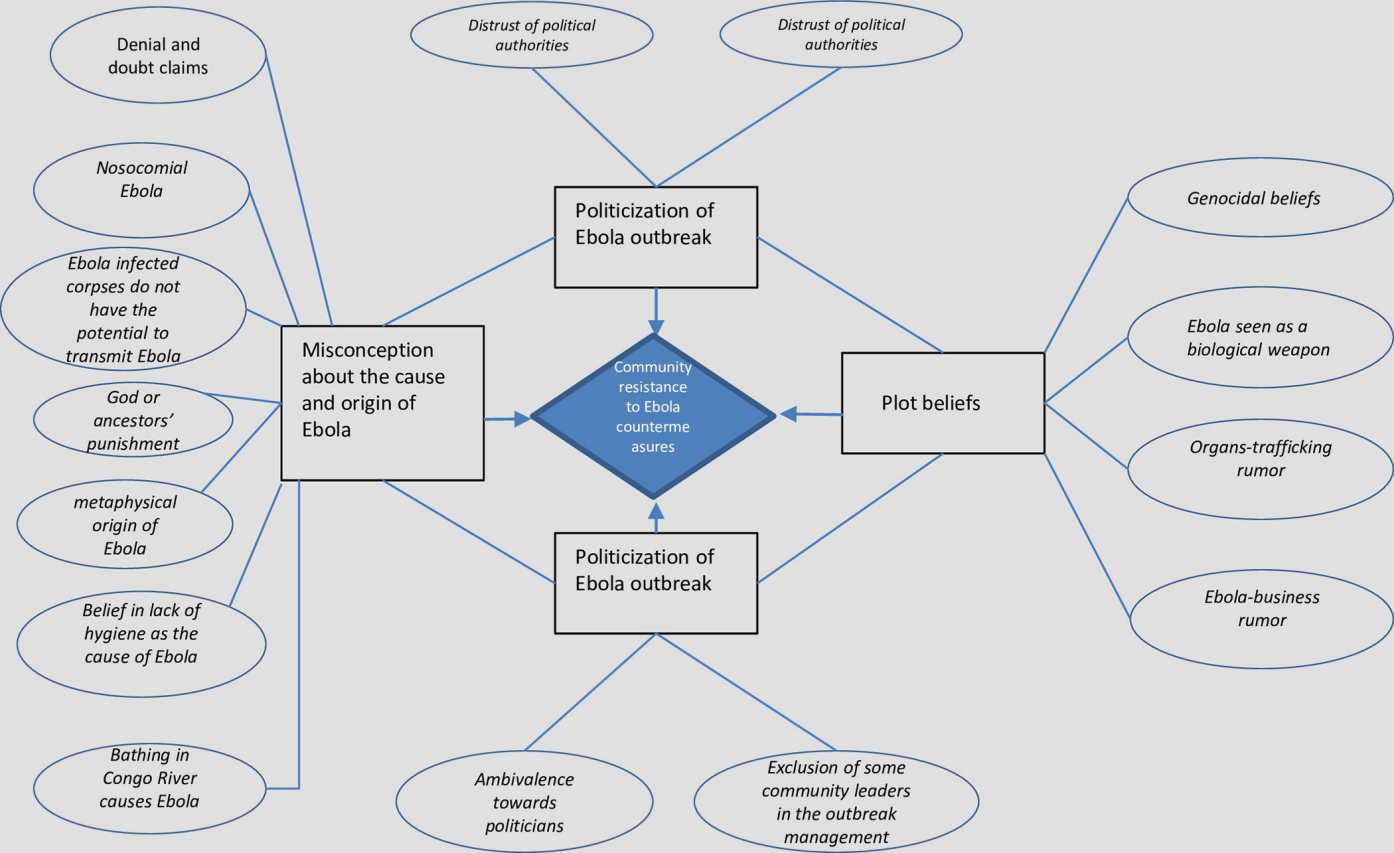
High-level themes (rectangular) and subthemes (oval) related to local views and beliefs on Ebola in DR Congo that lead to community resistance.

Participants’ responses produced the following 3 high-level themes and 15 subthemes regarding Ebola (Fig 2). The themes were inadequate knowledge of the causes of Ebola, Ebola seen as a plot by multinational corporations, and the politicization of Ebola. Most of these subthemes are described in previous reports and were merely confirmed in this study, but some were identified for the first time. Illustrative comments on the subthemes were identified.

### Theme I: Causes and transmission of Ebola

Participants were asked whether they had heard of the ongoing Ebola outbreak and to share what they believed were its causes and means of spread. Overall, their responses revealed excellent general awareness of Ebola. However, most had limited biomedical knowledge, and a difference was seen across areas. In the affected areas, the participants had better knowledge of the causes and modes of transmission. Most correctly identified Ebola as being caused by a human-transmitted virus. However, in the unaffected areas, although some participants identified the human-to-human mode of Ebola transmission, most indicated that there had been no awareness campaigns on the causes of Ebola. Many lacked adequate biomedical knowledge of the cause of Ebola or its modes of transmission. Most importantly, participants wished to know the origin of the outbreak. Their comments on the perceived causes of Ebola fell into seven subthemes (Fig 2). The first five were found in all regions: Belief in a metaphysical origin for Ebola, a lack of awareness of human-to-human transmission of Ebola via an infected corpse, the origin of Ebola in divine punishment, denial of Ebola’s existence, and a nosocomial origin for Ebola. In addition, the following two were reported only by participants from unaffected areas: poor hygiene and bathing in the Congo River.

#### Beliefs in a metaphysical origin for Ebola

Belief in a metaphysical origin was commonly found in all regional blocks and especially among pastors and among participants in the FGDs, who often endorsed the idea that the symptoms of Ebola come from witchcraft, curses, or bad lack. According to this belief, traditional healers and spiritual leaders are reliable for treatment of Ebola. Some participants insisted that if Ebola is caused by witchcraft, curses, or bad lack, going to a hospital to seek care would lead to death. For example, one participant stated that “Nowadays, witchcraft is using Ebola to kill people. If someone becomes economically successful, he needs supernatural protection to avoid being attacked by a sorcerer. Because of jealousy, a sorcerer can send you Ebola in a mysterious way. In that case, the victim should seek help from a traditional healer, and not from a hospital. A disease with no cure must be treated by a traditional healer.” However, many participants rejected this view.

#### Ebola viewed as divine punishment

Some pastors and FGD participants were convinced that prayers were a powerful tool that could stop Ebola, and they also believed that without prayer, the outbreak would not end. Some believed that people could contract Ebola from a demon or as a result of offending God. For example, one pastor said, “Our prayers are very strong and deep; therefore Ebola will never reach our church.” Participants also reported that “In the hot zones, some people who were advised to seek care from ETCs did not turn to ETCs. Instead, they visited pastors or spiritual healers.”

#### Ebola-infected corpses do not have the potential to transmit Ebola

Very few participants in the unaffected areas recognized that touching, bathing, and dressing an Ebola-infected corpse or attending a funeral of an Ebola victim have a high risk for Ebola infection. Some of them could not understand how even shaking hands at a funeral could lead to the spread of Ebola. For example, one participants in an unaffected area aid, “shaking someone’s hand at a funeral has nothing to do with Ebola transmission. Ebola is found in the east of DR Congo and is related to eating dead wild animals. Nothing else.”

Participants uniformly felt that not memorializing a dead person meant a failure to pay respect, and doing so could result in the return of the dead person’s spirit, which could bring harm to one’s family.

The reasons that the response teams provided for not touching the corpses were thought to be invalid and unfair. A man aid, “I could not touch my wife when she died from Ebola; the response team buried her against our wishes. This is simply unacceptable.”

#### Nosocomial Ebola

Several participants from the affected areas reported that local people thought that people contracted Ebola from health facilities. A teacher said, “It’s terrible here. I am afraid to seek care from a hospital when I have malaria symptoms such as a fever. If I go to the hospital, I may contract Ebola. Ebola used to be in animals in the forest, but now I hear that Ebola lurks in the hospital.”

#### Lack of hygiene as the cause of Ebola

This view was prevalent in the unaffected areas. Participants said that because people in the east of DR Congo have been living in war and poverty for years, water supply and sanitation might be a challenge. Most surveyed thought that these conditions enabled the Ebola outbreak. One participant said, “Listen, there are many displaced people and much insecurity in the Eastern DR Congo, hygiene is a problem, and that is why people there are getting Ebola.” Another participant commented, “I have my own latrine and I always wash my hands with soap after going to the toilet. I will never get Ebola.”

#### Bathing in the Congo River causes Ebola

This is a previously unreported rumor that had not been recognized until now. Only participants from the unaffected areas thought that you can be infected by Ebola by being in contact with the Congo River, such as when fishing, bathing, or drinking. A schoolteacher said, “In one fishing village located in Kongo Central (Bas-Congo), some fisherman died from Ebola like-symptoms. It means that they might have had Ebola by touching the Congo River water; investigation is needed for the fisherman who died.”

#### Denial and doubt

All participants reported hearing that Ebola does not exist in East DR Congo. At the beginning of the outbreak, they said, several people questioned the existence of an Ebola outbreak in the country, including political leaders. All of the participants believed that the Ebola virus was real. However, they reported that there are people in the country who doubted the existence of the outbreak. The following was a commonly expressed idea: “If Ebola is a real disease, why does it not kill the rebels, armed groups, or white foreign aid workers? Or why does this Ebola not cause hemorrhage in affected persons? This Ebola outbreak does not exist.”

One pastor asked for proof that Ebola was real. He said, “They promised us a safe and dignified burial. However, we still cannot touch our deceased relatives. So we throw stones at the Ebola team because they don’t bring proof to us that Ebola exists.”

Some participants stated that they had two responses to the question of whether Ebola exists in the Kivus. When a stranger asked, they acknowledged it. However, when the same question was asked by someone they knew, they would express doubt or deny it.

### Theme II: Ebola as a plot by multinational corporations

Participants were asked what they believed to be the ultimate origin of the ongoing Ebola outbreak. Their responses were grouped into four subthemes: genocide, biological weapons, organ trafficking, and business interests. These perspectives accused white foreigners, international pharmaceutical corporations, and oil companies, to name a few. The participants strongly expressed their belief that the virus might have been secretly fabricated by foreign governments. It was also viewed as a medical crime, due to the devastating mortality rate of this Ebola outbreak. Many participants believed that the Ebola virus was deliberately brought to the Kivus, and the experimental drugs and vaccines used in the outbreak were believed to sterilize people to depopulate the Kivus. They sometimes referred to earlier cases, such as in Iraq, where people suffered war without good reason.

#### Genocide

Most of the participants recognized that Ebola could have occurred naturally but the theory of a plot that Ebola was created to exterminate African black people was very prevalent. Some people refused to be vaccinated against Ebola because they considered it to be part of a genocidal campaign or because they feared becoming infertile. Participants believed that Ebola was develop to depopulate the Kivus, saying, “It is possible that Ebola was introduced on purpose to exterminate the local people of the Kivus. Ebola was brought into the Kivus to harm the people living there so white people could easily get access to minerals. We have watched many movies, and we believe in the Ebola plot.”

#### Ebola as a biological weapon

Some participants believed that Ebola was a bioweapon, made in a laboratory in a foreign country to harm people in the Kivus. There were also participants who argued that the length of this outbreak in the Kivus and the prevalent eating habits (that is, not hunting bats for food) supported their belief that Ebola was a bioweapon used to experiment on the inhabitants of the Kivus.

To argue for this point of view, one participant said, “How did Ebola spread in the Kivus when we know that eating fruit bats is not common there? My wife and I suspect that Ebola was fabricated and is being propagated on purpose.”

Another participant added that “Food habits in the Kivus are different from those in the Equateur province where Ebola first caused death. I think that Ebola has been weaponized to kill us for population control.”

During the FGDs, most participants nodded when these views were being expounded. In accord with this, vaccinations were seen by some to be an experiment conducted so that the US army would have a reliable vaccine in the future to protect its soldiers.

One political analyst said, “Imagine, without drugs against Ebola in Kikwit, Ebola would just last 3 months because all the sick people would die. However, with drugs and a vaccine, the epidemic takes a year. This is not normal Ebola.”

Another perception identified was that white people were testing drugs that they were preparing for a future biological attack. This participant said, “I heard that they are bringing medicine against Ebola in the Kivus to test it. Ebola had been manufactured at a Russian military lab, so they might attack the US at any time. To prepare themselves, the US is testing antidote medicines. Drugs against Ebola are not made primarily to help us, black people.”

Another participant said, “In Beni, people die for many reasons, including experimental Ebola drugs and vaccines. Some poisons are hidden in those experimental drugs.”

#### Organ trafficking

Many participants reported hearing about organ trafficking. They acknowledged that white people and other foreigners were often accused of spreading Ebola to harvest organs for trafficking with the help of local HCWs. They heard that some refuse to seek medical help because HCWs, who should treat patients, were in fact infecting people with Ebola, who were then killed, and then their organs were harvested and the body was filled up with concrete and buried. One participant commented that “It is difficult to know the truth; people are saying that if you go to ETCs, you will be injected with poison and your internal organs will be sold to white people.” Participants also reported that this rumor was reinforced by the observation of a high mortality in ETCs and the use of opaque plastic bags for burials without family members. One participant said, “I heard that most of the ETCs are built with cement barriers with barbed wire fences on the top. This might mean that something suspicious is happening in the ETCs.”

#### Ebola as a business

Despite Ebola’s high mortality rate, it was still seen as an opportunity to create jobs or make a profit. Several participants from across the country reported this subtheme. Some participants believed that international pharmaceutical companies, national health officials, and some frontline responders were making money from Ebola, not doing all they could to completely eradicate the outbreak by making a coordinated effort. Some participants also said that they had heard that national health officials in DR Congo were deliberately gaming the reporting system by fabricating data (for example, by increasing the number of Ebola cases reported) to attract funding from the international organizations. The participants described luxurious vehicles being used by Congolese health officials to visit work sites. They also reported that the Ebola outbreak had created jobs in the Kivus, where unemployment is high, and those benefiting from the outbreak wished for it to continue. For example, one religious leader said, “If you stop the source of money, you will see that this Ebola outbreak will stop too.” The participants also reported that it was common to the slogan “no Ebola, no job” from some Ebola responders.

Some participants described seeing Ebola vaccines being sold to local poor people in zones close to areas with intense Ebola outbreaks. However, one political leader denied that this was true.

### Theme III: Ebola and politics

The participants were concerned that the outbreak would worsen due to political influence. Some participants expressed satisfaction with the current regime and the way it was conducting intervention, most expressed dissatisfactions and concern about corruption among politicians. The participants accused the government that was in power in December 2018 of having politicized this outbreak.

#### Ambivalence of the political authorities

While fighting Ebola, politicians themselves contributed to the genesis of certain rumors. Most participants across DR Congo agreed that the ongoing outbreak was fabricated by politicians to destabilize the eastern part of the country. They reported that during the 2018 presidential elections, people from Beni and other areas were kept from voting for invalid reasons. They also felt that the Ebola outbreak was used as an excuse by the government to postpone elections in Beni and Butembo. One participant said, “They use lies to delay the general elections and they send security forces among the Ebola responders; it means that this Ebola outbreak has some political motivations.”

We also had participants who stated that some opposition leaders used Ebola to gain popularity and obtain seats in parliament. One teacher said, “[Our] national deputy said on social media that Ebola was not real, and if it was real, it was surely brought to the Kivus by malicious politicians. It is a manmade Ebola, which is being used for the extermination of local people in the Kivus.”

#### Exclusion of some community leaders

Some participants reported that local gatekeepers and other well-known Ebola responders were not involved in the response efforts from the beginning. One participant complained that “We cannot understand why the expert professor who ended Ebola in Yambuku is not part of the response. This is politics.”

Other participants considered that local Ebola educators who were involved in the response were not being well compensated compared to the many responders (who were seen as “foreigners” in the affected areas) from Kinshasa (and other African countries) and did not speak the local dialects. Some local people thought that these outsiders were sent by the former president to kill them using Ebola because they did not support him.

#### Distrust of the political authorities in DR Congo

The participants believed that the national authorities were selfish and were working to make profit for themselves and their families. They also expressed doubt regarding the efficacy of the healthcare system, considering that most public health priorities had been neglected for years by government authorities. For example, one teacher commented that “Ebola is just the ears of a hippopotamus or a symptom; the root causes are several challenges that the affected communities face, including extreme poverty, war, and the absence of a good public health system. We need to address their needs. But the government is not helping much.”

Interference with the presidential election and general poverty appeared to be the most important contributors to the distrust. The delay of the 2016 presidential election, which allowed the ruling party to remain in power until December 2018, contributed to this distrust. Few participants asserted their trust in the authorities. Many expressed frustration with the fact that many around them were experiencing socioeconomic hardship. One political analyst commented that “It is difficult for people to believe that the government will help them. For years, people in Eastern DR Congo have suffered from war and conflict, but nothing is being done.” They often repeated that distrust of the Ebola response team and the Congolese authorities was common. In addition, controversies among health officials regarding the use of a second vaccine against Ebola had even further eroded their trust in the health authorities. This was interpreted by some participants as evidence of a lack of motivation for the Congolese authorities to end the epidemic and was associated with financial opportunities for the Congolese authorities and pharmaceutical companies. One FGD participant said, “All these controversies surrounding the use of the second experimental vaccine make me think that the authorities and malicious white people want Ebola to make money while people are dying.”

Similarly, some participants were not confident in the government’s intention to eradicate Ebola or improve the health of the population. In addition, some reported reluctance to visit a healthcare facility because they felt that it was no safe. Participants from the Kivus in particular felt that for decades they had received little assistance from the healthcare system. One school teacher said, “Please see for yourself, measles is killing our children, but there is no help. We are very poor and the government has abandoned us. Our government thinks that we have the right to die but not the right to be alive.”

The majority of participants said that their bitter experience contributed to their distrust of the Congolese authorities. Most participants complained about how the security issue is being handled in the Kivus, and they believed that Congolese politicians are doing things that causing insecurity in the Kivus. Some said that the government has promised many times to bring security to the Kivus, but this has never been done. One participant said, “People do not trust our government; people compare our authorities to thieves, and that’s why we have doubts about all that they say to us.”

Some participants also doubted politicians’ integrity. One teacher said, “There are some ministers who invented Ebola to get money from the WHO. We now compare them to thieves.”

There were also participants who reported that they trusted the Congolese authorities because hoped that the socioeconomic situation would improve under the current ruling party.

#### Reduced trust in the healthcare system

Most participants said that due to accessibility, affordability, acceptability, and respect for patients, traditional healers were more trusted and more integrated than the formal health system, where HCWs are sometimes mistrusted by patients. They also felt that most patients in remote areas face challenges to obtaining medical treatment from the formal health system for common ailments, and participants considered that the interest of HCWs in financial gain was too high. One teacher said, “To go to the hospital, some nurses who are not kind will emphasize money. However, traditional healers do not put an emphasis on money and can cure Ebola. At the hospital, the probability of dying from Ebola is very high.”

Another participant gave the example that healthcare facilities were not furnished, and there was a lack of training for Ebola among some first-line HCWs: “How they will detect Ebola when they do not even have electricity at night or a thermometer? At our community’s dispensaries, nurses have not yet been trained to be able to detect suspected Ebola cases. Unbelievable.”

## Discussion

We carried out this nationwide qualitative research in this context of enhanced community skepticism of Ebola countermeasures and insufficient preparedness in areas that are unaffected by Ebola in DR Congo, one year after the beginning of the outbreak, when it was at its peak, to investigate rumors that were leading to community resistance or that might hamper Ebola response efforts in the unaffected zones. We solicited the views of locals, including religious leaders and teachers who may be among locally influential people, trusted and respected in their communities, as well as political leaders/analysts regarding the ongoing outbreak. Three themes and fifteen subthemes were identified from the FGDs and IDIs (Fig 2).

The findings showed that, the participants, even those with higher education, held unrealistic beliefs on the origin of Ebola in the Kivus. We also found that even political leaders/analysts and other highly educated participants believed in plot theories. Those who believe in the plots can endanger others by circulating rumors to their social media followers and potentially harming control efforts. Surprisingly, most of those who believed in Ebola as a plot did not give the impression that Ebola was a pressing problem, especially those who lived in the unaffected areas. This illustrates the need for better and more aggressive awareness campaigns in unaffected areas, bearing in mind that the health messages chosen should be adapted to the local context to improve public response [17]. Our study confirms previous reports on DR Congo that have collected rumors during this outbreak [7, 8, 18]. Because our findings are consistent with previous work, some differences can be seen among those reports that deserve highlighting. First, one study used quantitative methods to investigate rumors [7], and second, the three reports were limited to areas undergoing outbreaks. Rumors and skepticism were also prevalent in 2014 when Ebola spread in West Africa [19–22].

In this study, we also found that public trust in government was low even in unaffected areas, and there was a lack of general confidence in the Congolese authorities. This poses a significant challenge to prevention activities. This finding is in line with a previous study conducted in Liberia during an Ebola epidemic, which reported that those experiencing socioeconomic hardships were more likely to distrust the government [23] and another one, also conducted in Liberia, which reported that contact tracing was hampered by a lack of trust in government in the community [24]. However, our findings are in contrast with those of another study that found high trust in authorities in some populations during the West Africa Ebola outbreak [25]. Distrust of the national authorities may be a consequence of the beliefs in plots shown in this study. It could also be a consequence of various factors but can be attributed to a large degree to distal determinants, such as bitter experience of political instability and decades-long armed conflict in the Eastern DR Congo, unmet social needs, socioeconomic hardships, and an insufficiently developed healthcare system, among other questions. Distrust of authorities by locals may also be driven by the scarcity of effective government services across the country and the postponement and cancelation of the 2018 presidential election in Ebola-affected areas, including Beni City.

### Potential sources of rumors

Although other explanations are possible, one hypothesis is that in the unaffected areas, lack of awareness campaigns, and lack of access to reliable information on Ebola may have provided a fertile ground for rumors, disbelief, and gossip to grow. However, this may not imply that more extensive educational campaigns alone in these areas will be sufficient to change people’s perceptions; it may have only a limited effect on these rumors if social-cultural and political contexts are not also taken into account. Perhaps most importantly, correcting rumors alone can do additional harm [26]. Appropriate preparedness would involve an anthropological approach which incorporates dialog and listening to locals, which are crucial to enhancing engagement and participation in local populations; this would reinforce trust and curb Ebola rumors in unaffected areas.

In affected areas, a vicious circle might appear between rumors and distrust of the authorities and institutions among locals: decreased trust may facilitate the genesis of rumors and gossip, and the rumors could result in further erosion of confidence in authorities and institutions, along with non-compliance and misperceptions. In addition to this vicious circle, many other factors could explain some of the rumors found in this study in the affected areas. They include the deployment of the army to protect Ebola responders, the exclusion of some locals from the response efforts, and the way in which the response team carried out intervention. For example, it was not easy to be vaccinated; it was time-consuming, due to the requirement to verify people at risk. In addition, sometimes the team did not believe the information given and vaccination was not provided [18]. The poor leadership displayed by the response team at the beginning of the outbreak, the limited food support for the victims, weak human rights protections in the conflict zones, and international aid that did not benefit the locals also provoked the spread of rumors. Rumors were also fueled by public misconceptions of humanitarians and the national health authorities, who were considered to have financial motives, as well as suspicion of ETCs, which were considered to be places where Ebola could be contracted and a person could die, and the psychosocial dimensions of the disease, where some people felt that their traditional culture and customs were not being respected.

### Consequences of rumors

The findings of this work support those of a previous study conducted in Ghana [19], which reported several misconceptions of the modes of transmission and management of Ebola. These rumors and decreased trust in the affected areas led people to be afraid and anxious. They expressed their anxiety by resisting the efforts of Ebola response teams. We identified many compensatory strategies that people employed to express resistance. They included hesitation on the part of people with Ebola symptoms to seek care from health services or ETCs during the epidemic. People were advised to visit traditional healers and did not survive. However, it is important to note that it is common for Congolese and African people to seek help from traditional healers because in some cases they constitute the only affordable health service, especially in remote settings [27]. The consequence of beliefs in Ebola plots led in two directions. Despite their beliefs in plots, well-educated participants who endorsed plot beliefs were likely to engage in prevention measures and were very supportive of care-seeking behaviors. However, those who reported beliefs in plots tended also to say that they did not engage in preventive measures or care-seeking behaviors, as previously reported by Bunduki [9]. This in line with a US study that highlighted that participants who believed in conspiracy theories were less supportive of Ebola countermeasures or policies such as quarantine [28].

### Implications for policy makers

The findings of this study are of particular interest because they shed light on the beliefs of local people concerning Ebola. The identification of these rumors can serve as a gateway for understanding local beliefs and they can be incorporated help to fight Ebola. Participants’ health behaviors, including community resistance, are the consequences of their beliefs about Ebola. One pragmatic implications of these findings is that the rumors identified in this study are useful for advocating and guide aspects of preparedness, such as public health education on Ebola in the unaffected areas, while acknowledging that no easy solutions exist to overcome the distrust.

Our findings are a reminder that the Ebola outbreak in DR Congo is not a medical problem alone but a socio-cultural one as well. To understand what it implies if Ebola is socio-cultural problem, it is important to note that the medical response to Ebola interferes with local practices (such as preventing traditional caring for the sick and burial practices) in a context where there is already a lack of trust in leaders and a battered health system, compounded by a humanitarian crisis due to civil unrest and conflict [29]. Providing adequate health services and obtaining access to them is a challenge for many Congolese even in the best of circumstances [30]. It is not rare to find that healthcare facilities are enabling Ebola transmission in DR Congo, such as when a bed previously occupied by an Ebola patient is used by a new patient without being cleaned, as occurred in the 1970s [31]. Therefore, the health system of DR Congo must be strengthened and its resilience improved to respond to future volatility situation. Initiatives to address socioeconomic problems are also needed: for instance, many people lack tap water and soap, but they are nevertheless advised to wash their hands frequently. Infectious diseases, such as typhoid, which attracts less media attention, should also be targeted, as Médecins sans Frontières did in North Kivu [3]. We argue that a more holistic approach of health is needed [32] as a long-term priority. During an Ebola crisis, global health organizations should also invest in social response, including devoting energy to addressing food security, drinking water, education, and other diseases, including malaria. This view is supported by a study in Sierra Leone, which suggested that Ebola in Africa should be “re-contextualized and interpreted within a broader framework of health concern for all people” [22]. This also suggests that medical intervention alone is insufficient. We need to recognize the importance of coordinating and cooperating with the general population, in addition to anthropologists, politicians, the media, religious leaders, and private sector allies, to name just a few, as this could help lower the fear and anxiety associated with Ebola.

### Study limitations

Although the participants were recruited from the entirety of DR Congo and our findings are likely illustrative of widely held beliefs, they may not accurately represent the entire population. For instance, some rumors or misperceptions about Ebola may have been missed. Only 10 participants were from the Kivus, and all FGD participants were from Kinshasa and Bas-Congo, far from the outbreaks. Thus, our results may be regionally biased, as attitudes toward Ebola may change after direct experience of the disease [22, 27]. This lack of coverage of views in Ebola-affected areas is a limitation undermining the generalizability of our findings beyond the regions explored. Even though our results cannot be generalized to the entire DR Congo, they should not be interpreted as meaning that people in outbreak areas were not skeptical about measures to protect themselves from Ebola [18]. Furthermore, only participants who were interviewed face-to-face had the opportunity to listen and review their statements. Those who were interviewed over the telephones and those who participated in the FGDs were not given this opportunity. We cannot rule out the possibility that some participants’ responses were misinterpreted. However, this is mitigated by the fact that concerns over translation accuracy can be ruled out, as the principal investigator (BAM) and some study team members are fluent in both French and Lingala, the two languages used to collect data, and the interview notes were carefully reviewed together with the audio transcriptions to prevent any meaning from being lost from the original statements.

In addition, we were unable to verify whether the rumors identified in this study were true. For example, some claims, such as how vaccines were being sold to the poor, could not be verified. In addition to these limitations, this study has some strengths. First, the opportunities that its findings offer to identify misperceptions of the Ebola outbreak are paramount. Second, this was a nationwide study where participants were recruited from both affected and unaffected areas, a definite strength.

## Conclusions

Between August and September 2019, rumors were widespread, and confidence in authorities was low, in both affected and unaffected areas. The consequences for the affected areas were community resistance to interventions, with low adherence to Ebola countermeasures, making it difficult to conduct contact tracing, to treat people at the hospital and thus dying, to deliver medication or vaccines against Ebola, and to change behavior in at-risk populations (such as unsafe burial and funeral practices). These circumstances likely increased the spread of the Ebola virus. The participants’ comments in this study suggest that dialog with local communities in need of support should continue, and those who speak local languages should be included in the response team.

Insufficient preparedness in areas that have not been affected by Ebola helped fuel the rumors. For example, the rumor that Ebola does not exist was common in unaffected areas, as participants believed that it was just an excuse to exclude people from voting in the presidential election to postpone the 2018 election. We therefore also recommend health education and awareness campaigns and that the priorities of the population (including jobs and health) be prioritized, as well as taking action to strengthen the health system for sufficient preparation for future outbreaks. The findings remind Ebola intervention teams that controlling rumors and gaining public trust are important to ensuring that the Ebola response is more effective. The results of this study highlight that the healthcare system in the DR Congo must be better supported, in tandem with attention being paid to security issues and political instability; this could help avoid future outbreaks. Due to the skepticism against Ebola countermeasures, understanding what is being thought about Ebola is critical before taking action, and this includes assessment that can integrate the unmet needs of the population.

## Supporting information

S1 Appendix (DOCX)

S2 Appendix (DOCX)
