# Supplementary material for: Local perspectives on Ebola during its tenth outbreak in DR Congo: A nationwide qualitative study
Source: PLoS One. 2020 Oct 22;15(10):e0241120. doi: 10.1371/journal.pone.0241120 (PMC7580967; doi:10.1371/journal.pone.0241120)
Supplement: S1 Appendix — (DOCX) [file pone.0241120.s001.docx]

**Titre de l’étude:** Enquête sur les connaissances, attitudes et pratiques sur la maladie à virus Ebola en République Démocratique du Congo (RDC).

1. **Guide d’entretien individuel pour l’étude qualitative sur la maladie à virus Ebola en RDC.**

**(NB: Ce questionnaire doit être administré à ceux qui sont âgés de 18 ans et plus).**

#### **Nom de l’enquêteur:**

#### **Date de l’entretien:** __ __ / __ __ / __ __ __ __

(Jour) (Mois) (Année)

Heure de démarrage: __ h __m

Heure de la fin: __ h __m

**Pour commencer, je voudrais vous poser quelques questions sur vous-même. Je vous rappelle que ces informations sont confidentielles et ne seront utilisées que pour les besoins de l’enquête**

1. **Identification et informations démographiques**

| **No.** | **Questions** | **Catégories et codes** |  |
| --- | --- | --- | --- |
|  | Cité ou ville |  |  |
|  | Province (Cocher sans demander au participant) |  |  |
|  | Consentement éclairé | Signature obtenue 1  Refus 2 | **🡪Arrêter l’interview** |
|  | Quel âge avez-vous? (en ans) | Age (Année): |  |
|  | Genre (Cocher sans demander au participant) | Home 1  Femme 2 |  |
|  |  |  |  |
|  | Niveau d’étude | N’a pas fini l’école primaire 1  A fini l’école primaire 2  A fini l’école secondaire 3  A fini l’université 4 |  |
|  | Emploie |  |  |

1. **Connaissances sur la maladie à virus Ebola**
2. Que ce qui est à la une en RDC concernant la sante? Surtout à la partie Est de la RDC.
3. Est-ce nous pouvons parler un peu de la maladie à virus Ebola qui sévit aux Kivu? Qu’en ce que vous en savez?
4. Croyez-vous que Ebola existe en RDC? Quel est votre point de vue?
5. Quelle est la cause de Ebola?
6. Quelle est d’après vous l’origine de cette épidémie à virus Ebola?
7. Pourquoi les gens souffrent de Ebola? Pensez-vous que Ebola peut arriver ici/dans votre milieu?
8. Comment se transmet Ebola? Pourquoi assister à des obsèques peut-il constituer un risque de contamination si le défunt a souffert de Ebola ?
9. Quelle est la période de l’incubation de Ebola? (Je vous rappelle que la période de l’incubation est la période de temps entre l'infection par le virus Ebola et l'apparition des premiers symptômes)
10. Quels sont les signes qui montrent qu’une personne souffre de Ebola?
11. **Attitude dans la prévention concernant la maladie à virus Ebola**
12. Pourquoi d’après vous certaines personnes qui souffrent de Ebola refusent d’aller à l’hôpital?
13. Comment les gens qui souffrent de Ebola sont vus par les autres dans la communauté ?
14. Comment on se protégé contre Ebola?
15. Comment éviter Ebola? Quelles sont les précautions à prendre?
16. Pourquoi les gens qui traitent Ebola portent des masques ? souhaiteriez-vous en porte un pour vous protéger ?
17. Si par exemple vous attrapez Ebola ou si quelqu’un dans votre famille étroite est suspect d’Ebola, que feriez-vous? Irez-vous consulter un infirmier ou un docteur?
18. Que feras tu si un membre de famille étroite contacte Ebola ?
19. **Attitude envers le gouvernement**
20. Quelle est ton avis concernant l’actuel gouvernement?
21. Pensez-vous vous que les autorités pensent à la population?
22. Avez-vous confiance en notre gouvernement concernant leur façon de lutter contre Ebola?
23. Avez-vous confiance en nos médecins, et infirmiers concernant leur capacité de traiter Ebola?
24. **Barrières et solutions possibles pour mettre fin** à **cette épidémie**
25. Pourquoi cette épidémie tarde à être contrôlé?
26. Quel est votre avis sur le vaccin contre Ebola? Accepteriez-vous d’être vacciné?
27. Que ce qui peut pousser les chefs de communautés ou religieux à s’engager vraiment dans la lutte contre Ebola?
28. Autre commentaire sur l’épidémie à virus Ebola dans notre pays. Si vous aviez des suggestions à faire à propos de cette épidémie, quelles seraient-elles ? Et ça sera votre mot de la fin.

Nous vous remercions beaucoup pour votre temps.

**Translation of the in-depth interview guide from French (original) to English**

Title of the study: Survey on knowledge, attitudes and practices on Ebola virus disease in the Democratic Republic of Congo.

1. Individual interview guide for the qualitative study on Ebola virus disease in DR Congo.

   (NB: This questionnaire should be administered to those aged 18 and over).


   Name of investigator:
   Date of interview: __ __ / __ __ / __ __ __ __
    (Day month Year)
   Start time: __ h __m
   End time: __ h __m

To begin with, I would like to ask you a few questions about yourself. I remind you that this information is confidential and will only be used for the purposes of the investigation

I. Identification and demographic information

| **No.** | **Questions** | **Category and codes** |  |
| --- | --- | --- | --- |
|  | City/town |  |  |
|  | Province (Check without asking the participant) |  |  |
|  | Informed consent | Obtained 1  Refusal 2 | **🡪Stop** |
|  | How old are you? (in years) | Age (Year): |  |
|  | Gender (Check without asking the participant) | Male 1  Female 2 |  |
|  |  |  |  |
|  | Educational level | Did not finish primary school 1  Primary school 2  Secondary school 3  College 4 |  |
|  | Employment/job |  |  |

II. Knowledge about Ebola virus disease

9. What is in the news in DR Congo concerning health? Especially in the eastern part of DR

Congo.
10. Can we talk a bit about Ebola virus disease? What do you know about it?
11. Do you believe that Ebola exists in the DR Congo? What is your point of view?
12. What is the cause of Ebola?
13. What do you think is the origin of this Ebola virus epidemic?
14. Why do people suffer from Ebola? Do you think Ebola can happen here / in your community?
15. How is Ebola transmitted? Why can attending a funeral constitute a risk of contamination if the deceased suffered from Ebola?
16. What is the incubation period for Ebola? (I remind you that the incubation period is the period of time between infection with the Ebola virus and the appearance of the first symptoms)
17. What are the signs that a person has Ebola?

III. Attitude towards prevention regarding Ebola virus disease

18. Why do you think some people with Ebola refuse to go to hospital?
19. How are people who suffer from Ebola seen by others in the community?
20. How do you protect yourself against Ebola?
21. How to avoid Ebola? What precautions should be taken?
22. Why do people who treat Ebola wear masks? would you like to wear one to protect yourself?
23. If, for example, you got Ebola or someone in your close family is suspected of having Ebola, what would you do? Will you go see a nurse or a doctor?
24. What will you do if a close family member contacts Ebola?


IV. Attitude towards government

25. What is your opinion of the current government?
26. Do you think the authorities think of the people?
27. Do you trust our government in their way of fighting Ebola?
28. Do you trust our doctors and nurses in their ability to treat Ebola?

V. Barriers and possible solutions to end this epidemic

29. Why is this epidemic taking so long to be controlled?
30. What is your opinion on the Ebola vaccine? Would you agree to be vaccinated?
31. What can push community or religious leaders to really get involved in the fight against Ebola?
32. Do you have any other comment on the Ebola virus epidemic in our country. If you had any suggestions for this epidemic, what would they be? And that will be your final word.


Thank you very much for your time.
